# High-flow nasal cannula for body rewarming in hypothermia

**DOI:** 10.1186/s13054-020-2839-1

**Published:** 2020-03-30

**Authors:** Emanuele Gilardi, Martina Petrucci, Luca Sabia, Kidane Wolde Sellasie, Domenico Luca Grieco, Mariano Alberto Pennisi

**Affiliations:** 1 Scienze dell’emergenza, anestesiologiche e della rianimazione, Fondazione Policlinico Universitario A. Gemelli IRCCS, L.go F.Vito, 00168 Rome, Italy; 2 Istituto di Anestesiologia e Rianimazione, Università Cattolica del Sacro Cuore, Rome, Italy

**Keywords:** High-flow nasal cannula, Hypothermia

Dear Editor,

Use of high-flow nasal cannula (HFNC) is common in critically ill patients with or at risk of respiratory failure. Its benefits include accurate delivery of the set fraction of inspired oxygen (FiO_2_), carbon dioxide washout from nasopharyngeal dead space, provision of small degree of positive end-expiratory pressure, and improved tolerance due to the comfortable interface [1–3]. To continuously deliver flows up to 60 l/min, inspired gas is actively conditioned through a heated humidifier, which increases gas temperature and absolute humidity up to 37 °C and 44 mgH_2_O/l.

Airway warming (i.e., respiratory insulation) is a technique previously described to treat hypothermia [4]. In the lungs, the surface available for heat exchange is that of pulmonary havens, with a total area of about 100–140 m^2^. Moreover, inhalation of heated air yields vasodilation of alveolar capillaries, which further increases the surface for heat exchange between the blood and alveolar gas. Full humidification of inhaled air enhances heat transfer and conduction [4, 5].

Over a 6-month period (October 2018–March 2019), we applied, for clinical purposes, HFNC with no oxygen supplementation to 4 patients (3 females, median [interquartile range] age 51 [67–86] years), who were admitted to the emergency department of our institution with stage 1–2 primary hypothermia (i.e., prolonged exposure to cold environment) [6]. All patients were fully awake, hemodynamically stable, and had no respiratory distress nor gas exchange impairment. HFNC was administered through the AIRVO 2 device (Fisher and Paykel healthcare, New Zealand) or by a gas-compressed mechanical ventilator (EvitaXL or EvitaInfinity, Draeger, Lubeck, Germany) through a heated humidifier (MR860, Fisher and Paykel Healthcare, New Zealand): gas flow was set at 50–60 l/min, humidification chamber at 37 °C, and FiO_2_ at 21%. In all subjects, body temperature was recorded every 15 min through a dedicated urinary catheter (Teleflex, Annacotty, Limerick, Ireland). We retrospectively compared these patients with 4 matched control subjects (2 females, median [interquartile range] age 70 [52–80] years) who were admitted to the emergency department due to primary hypothermia in the same time period, did not receive HFNC, had no respiratory failure, and had body temperature recorded with the same technique: 1:1 matching was performed solely on the basis of body temperature at admission ± 0.2 °C. As a standard of care in our institution, all patients received treatment with warm blankets and heated crystalloid infusion, and the treatment was continued to achieve a core body temperature of 36 °C. All patients provided informed consent to data analysis and publication.

The median [interquartile range] body temperature at admission was 32.4 [32–32.9] °C in both groups. In the initial 5 h of treatment, the median crystalloid infusion was 3.3 l [2.6–3.8] in patients treated with HFNC and 3.3 l [2.3–4.3] in control subjects. The median time to rewarming (defined as sustained body temperature ≥ 35 °C) was shorter in patients treated with HFNC: 120 [120–165] versus 345 [218–405] minutes (Mann-Whitney *p* = 0.026). In the initial 5 h of treatment, the body temperature was significantly higher in HFNC patients than in the control group: the mean inter-group difference was 1.5 °C [95% confidence interval, 0.7–2.2] (repeated measures ANOVA *p* = 0.003) (Fig. 1). Consistently with the broad safety spectrum and device tolerability of HFNC [1], no treatment-related side effects were observed.

**Fig. 1:**
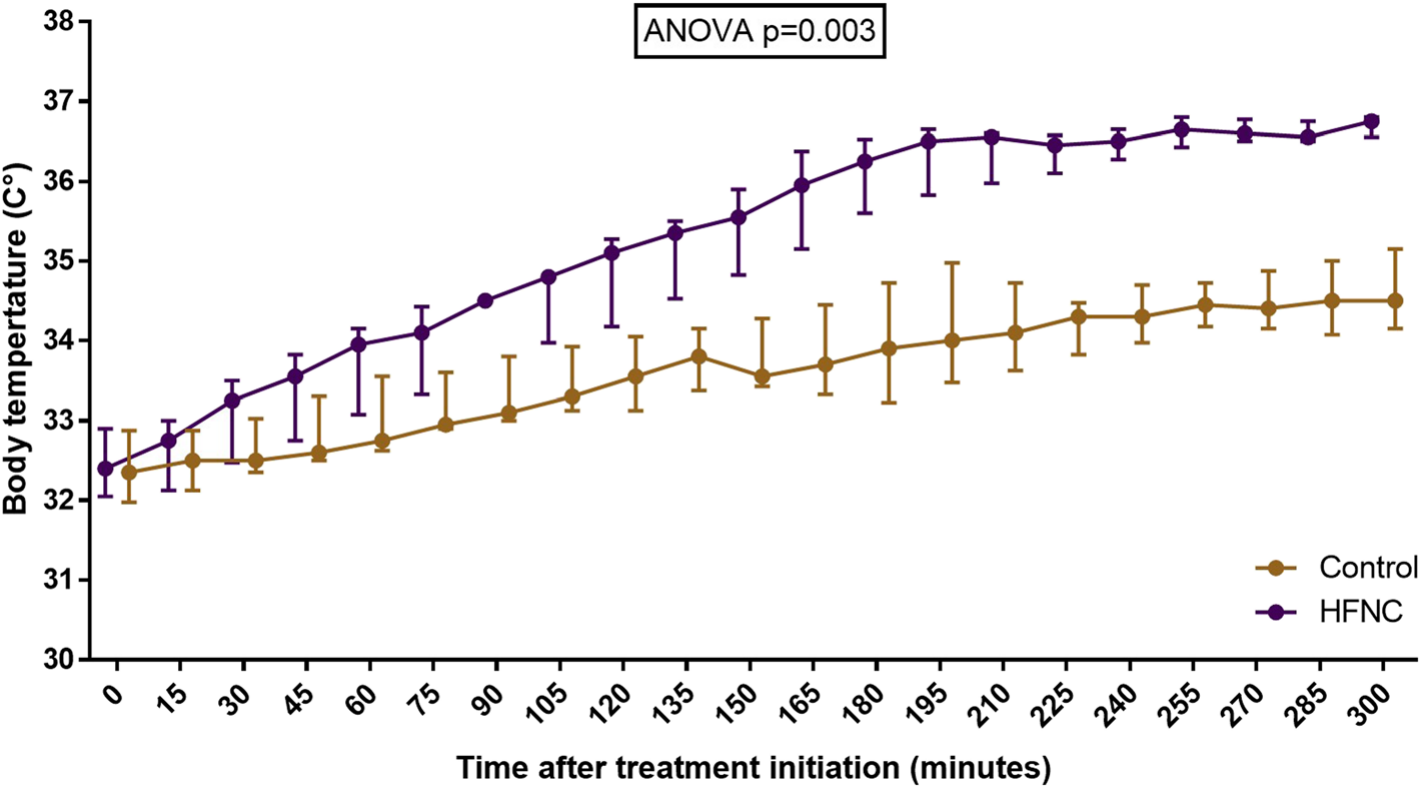
Body temperature in patients treated with HFNC and in control subjects. Medians and interquartile range are displayed for each time point. Repeated measures ANOVA two-sided *p* is reported

Despite the non-randomized design of our study and the limited sample, this preliminary report suggests that heated air administration through HFNC may represent an easy-to-use tool for respiratory insulation in patients with stage 1–2 hypothermia and no signs of organ dysfunction, independently from the presence/risk of respiratory failure and gas exchange impairment.

## Data Availability

The datasets used and/or analyzed during the current study are available from the corresponding author on reasonable request.

## Abbreviations

HFNC: High-flow nasal cannula
FiO_2_: Fraction of inspired oxygen
